# Associations between socioeconomic, parental and home environment factors and fruit and vegetable consumption of children in grades five and six in British Columbia, Canada

**DOI:** 10.1186/1471-2458-14-150

**Published:** 2014-02-11

**Authors:** Adrienne Attorp, Jenny E Scott, Ann C Yew, Ryan E Rhodes, Susan I Barr, Patti-Jean Naylor

**Affiliations:** 1 School of Exercise Science, Physical and Health Education, University of Victoria, PO Box 3015, STN CSC, Victoria, BC V8W 3P1, Canada; 2 Faculty of Health Sciences, Simon Fraser University, Blusson Hall, Room 11300, 8888 University Drive, Burnaby, BC V5A 1S6, Canada; 3 Department of Biomedical Physiology and Kinesiology, Simon Fraser University, 2600, 515 West Hastings St, Vancouver, BC V6B 5 K3, Canada; 4 Food, Nutrition and Health, University of British Columbia, 2205 East Mall, Vancouver, BC V6T 1Z4, Canada

**Keywords:** Child health, Socio-economic status, Fruit and vegetable consumption, Canada

## Abstract

**Background:**

Regular fruit and vegetable (FV) consumption has been associated with reduced chronic disease risk. Evidence from adults shows a social gradient in FV consumption. Evidence from pre-adolescent children varies and there is little Canadian data. This study assessed the FV intake of school children in British Columbia (BC), Canada to determine whether socio-economic status (SES), parental and the home environment factors were related to FV consumption.

**Methods:**

As part of the BC School Fruit and Vegetable Nutrition Program, 773 British Columbia fifth-and sixth-grade school children (Mean age 11.3 years; range 10.3-12.5) and their parents were surveyed to determine FV consumption and overall dietary intake. Students completed a web-based 24-hour dietary food recall, and a student measure of socio-economic status (The Family Affluence Scale). Parents completed a self-administered survey about their education, income, home environment and perceptions of their neighbourhood and children’s eating habits. Correlations and multiple regression analyses were used to examine the association between SES, parental and home environment factors and FV consumption.

**Results:**

Approximately 85.8% of children in this study failed to meet minimum Canadian guidelines for FV intake (6 servings). Parent income and education were not significantly associated with child FV consumption but were associated with each other, child-reported family affluence, neighbourhood environment, access to FV, and eating at the table or in front of the television. Significant positive associations were found between FV consumption and child-reported family affluence, meal-time habits, neighbourhood environment and parent perceptions of the healthiness of their child’s diet; however, these correlations were weak (ranging from .089-.115). Multiple regression analysis showed that only child-reported family affluence significantly predicted FV consumption (std-β = 0.096 95% CI = 0.01 to 0.27).

**Conclusions:**

The majority of children in our study were not meeting guidelines for FV intake irrespective of SES, parent perceptions or home environment, making this a population wide concern. An almost trivial socio-economic gradient was observed for the child-reported SES measure only. These results are consistent with several other studies of children. Longitudinal research is needed to further explore individual and social factors associated with FV consumption in childhood and their development over time.

## Background

Globally and in Canada, the prevalence of obesity and other nutrition-related risk factors for chronic disease have been climbing in both children and adults [1-6]. The fruit and vegetable food group is widely recognized as crucial to promoting healthy weights and preventing chronic disease [7]. Epidemiological evidence suggests that regular fruit and vegetable consumption is associated with a significant reduction in the risk of chronic diseases such as cancer, cardiovascular disease, and obesity [8-12]. Thus, understanding the factors that influence their consumption is vital to the health of the population.

A large body of evidence indicates that socio-economic status (SES) plays a significant role in the diet of adults [13-19]. The results from research conducted on the relationship between SES (largely parental income and education) and the diet of children (pre-adolescents) and youth (adolescents) are less definitive. It could be expected that because most children and youth live at home with either a parent or a guardian, many of the disparities in health and nutrition that exist between low and high SES adults would translate to their children. However, some studies have failed to show these relationships while others have, although these effects are to a much smaller degree in these populations than in adults [20-22].

For example, in the case of youth (adolescents) Riediger and colleagues conducted a cross-sectional study of Canadian adolescents using data from the Canadian Community Health Survey, Cycle 2.1. They found the proportion of adolescents consuming the recommended amounts of fruit and vegetables increased from 34.2% in the lowest categories of household income to 42.1% in the highest categories (Odds Ratio = 1.07, p < 0.001) [23]. As well, positive associations were found between household education level and fruit and vegetable consumption (Odds Ratio = 1.12, p < 0.001). Although studies conducted outside of Canada have shown a positive relationship between SES and nutrition in pre-adolescent children [24-26], there is limited information on Canadian children. Researchers have identified both that social factors associated with fruit and vegetable consumption may differ for adolescents and children, and that there was a need for studies involving populations from different political contexts [27].

Beyond the socio-economic (income and education) determinants a number of other social factors have been associated with healthy eating in children. These include gender, culture, family (parental modeling, family structure, parenting style, meal structure – family meals, positive attitudes/parental support for healthy eating), exposure to media - in particular television, watching TV while eating, and home and school availability and access to healthy food choices [4,28,29]. Taylor, Evers and McKenna (2005) emphasized a need for studies on both the determinants of, and children’s eating behaviour, in Canada [4].

Thus the purpose of this research was to examine the relationship between key social determinants and fruit and vegetable consumption of pre-adolescent children (10-12 years of age) in British Columbia, Canada. Specifically, we examined the relationship between socio-economic status, parent and home environment factors and fruit and vegetable consumption in the past 24 hours.

## Methods

### Study design

We conducted a cross-sectional descriptive analysis using baseline data from the British Columbia School Fruit and Vegetable Nutritional Program (SFVNP) study, a representative matched comparison trial evaluating the effectiveness of delivering local fruit and vegetable to classrooms twice a week in alternate weeks. Specifically, we examined the relationship between socio-economic status, parental and home environment factors and fruit and vegetable consumption in 1421 grade 5 and 6 children (mean age = 11.3 years; range 10.3-12.5) attending 20 schools across three regions. Baseline data were collected during the winter of 2008.

### Participants

Participants in this study were fifth and sixth grade children and their parents recruited through elementary schools (n = 20) within sixteen British Columbia (BC) school districts that agreed to participate in the study. Students came from one of two school cohorts: a) schools that had registered to participate in the School Fruit and Vegetable Nutritional Program (SFVNP) in the upcoming year and had not implemented or received training about any other current elementary school healthy eating or healthy weights initiatives (n = 33), and b) schools that weren’t registered in the SFVNP, were located in the same geographic areas and also had not been exposed to other healthy eating/weights initiatives (n = 44). Ten SFVNP (Response rate of 30%) and 10 comparison schools (Response rate of 22%) agreed to participate. The average student response rate within these schools was 51% (n = 773) and the parental response rate was 49% (n = 737).

### Data collection procedures

The University of Victoria Human Research Ethics and the University of British Columbia Behavioural Research Ethics Boards, school boards and school administrators approved the study. Parent and student consents were distributed and collected by teachers prior to data collection. Student questionnaires were administered in the school computer lab. Children were asked to work individually but could seek help from a research team member if needed. Parent surveys were completed at home and returned to the school in a sealed envelope. Each instrument is described following.

### Measurement

#### ***Dietary intake***

To measure dietary intake, we used a web-based 24-hour dietary recall that asked students to report their food and beverage intake on the previous day. In all but one school, testing was conducted Tuesday through Friday, to assess weekday intake. The survey, which has been described elsewhere [29] and validated for this age group [30,31], was designed to minimize recall error and inaccurate estimation of portion size in younger populations by using built-in prompts and visual aids to reduce abstract thinking. In the validation study with Grade 6-8 children there was good agreement for energy and key nutrient intakes when compared with dietitian-administered food recall interviews for the same 24-hour period [30].

Foods were classified as part of the four main food groups from the current Canadian food guide “Eating Well with Canada’s Food Guide” [32]: fruits and vegetables, grain products, milk and alternatives, meat and alternatives. Foods not included in these groups were classified as “other”. Mixed meals were broken down into their component foods appropriate to the serving sizes and based on standard foods listed in the Canadian Nutrient File database [33]. Juice was classified as a fruit or vegetable depending on content (e.g. carrot versus orange) and potatoes and French fries were classified as vegetables. Upon completion of the survey, each student’s intake was summed and an analysis of food groups and macro and micro-nutrients (of approximately 500 foods) was conducted using ESHA Food Processor [34] and the 2007b Canadian Nutrient File [33].

#### ***Socio-economic status (SES)***

To measure SES we collected data from both the child and their parent. At the child level we used the family affluence scale (FAS) from the Health Behaviour in School Children study [35] that is comprised of four questions about family car ownership, number of computers in the household, number of family holidays, and whether or not the child had his/her own bedroom. The scores on these four factors are then summed to provide a continuous affluence variable with possible scores ranging from 0 to 9 [35]. The FAS is considered easy to complete and an accurate, non-sensitive method of addressing the issue of material affluence in children’s surveys [35]. In contrast to parental occupation, the proportion of missing data on FAS items in the validation study was low, and FAS items validated against parental responses showed strong agreement. In addition, FAS aggregated at a country level against Gross Domestic Product (the indicator of national wealth), indicating good criterion validity [35].

Second, the parent survey, adapted from the REAL Kids Alberta study [36] asked a parent to complete 32 questions which included questions about their current household income, education, gender, and place of birth (Canada or other). Highest level of education attained and income were reported on ordinal scales (education = elementary, secondary, community/technical college, university and graduate university, and income = less than $25,000, $25,000-$50,000, $50,001-$75,000, $75,001-$100,000, and more than $100,000).

#### ***Other social factors***

In addition to the above demographic information we assessed other social factors using the REAL KIDS parent survey [36]. For this study, we were interested in the following items related to FV intake: neighbourhood environment, access to fruit and vegetables, family dining behaviours (in front of the television, at the table, or eating out), parents’ perception of whether their child’s eating behaviours/habits were healthy, and how much they personally cared about eating healthy foods and exercising (all on 4 point likert scales).

The neighbourhood environment score was generated by summing 8 scores (4-point likert scale with 1 = strongly disagree and 4 = strongly agree) on previously validated items about neighbourhood [37-39] including items that addressed: a) neighbourhood satisfaction and services (liking where they live, access to recreation programs and facilities and access to stores to purchase fresh fruits and vegetables); b) neighbourhood safety (safety to play outdoors during the day, safety of children related to crime and traffic); and c) neighbourhood parks and playgrounds (presence of parks, playgrounds and places to play, presence of sidewalks on most streets).

The access to stores question from the neighbourhood measure was used as an independent assessment of a supportive neighbourhood fruit and vegetable environment. The family dining questions were from the Harvard Food Frequency Youth Adolescent questionnaire [40] and the parent’s perception of their child’s eating and their personal caring about healthy eating and physical activity and PA were developed, piloted and adjusted by the REAL KIDS Alberta survey team for use with the parents of pre-adolescent children [41].

### Data analysis

Data were entered into an Excel spreadsheet and statistical analysis of the data was conducted using the software program Statistical Package for Social Sciences version 16.0 (SPSS). Means and standard deviations were generated for key descriptors, and point biserial (*r*_*pb*_) or Spearman’s correlations (*r*_*s*_) were used to analyze relationships amongst the variables depending on the measurement scales used. Using matched parent and child data, we then constructed a multiple regression model to explore which of the significantly correlated variables predicted fruit and vegetable consumption. We used Cohen’s indices [42] to describe effect sizes.

## Results

In all, 312 boys and 353 girls completed the web based 24-hour dietary food recall at baseline. Those with high reported food group intakes (more than 3 standard deviations from the mean) were removed from analysis (FV >10.5, grain products >14.8, milk and alternatives > 6.8, meat and alternatives > 6.1, other > 11.2), resulting in a sample size of n = 597. Means and standard deviations for baseline intakes of food groups are presented in Table 1, which shows that mean servings were below recommendations for Vegetables and Fruit and for Milk and Alternatives, and approximated recommendations for Grain Products and Meat & Alternatives. The mean reported FAS was 5.98 (SD = 1.74) out of a maximum of 9. The mean neighbourhood environment score was 25.25 (SD = 3.26) out of a maximum of 32. Key demographic and other social factor descriptors for parents of participating children are displayed in Tables 2 and 3, respectively. Based on recommendations outlined in the 2007 Canadian food guide [32], 85.8% of participants failed to meet the guidelines of six daily servings of vegetables and fruit.

**Table 1:** Descriptive statistics for food group and energy intake of BC children in grades five and six (n = 597)

**Recommended food group servings**^**1**^	**Mean servings (SD)**	**Median**	**Range (servings)**
Vegetables and fruit (6)	3.33 (2.42)	2.9	0 -10.5
Grain products (6)	5.97 (2.84)	5.6	0- 14.8
Milk & alternatives (3-4)	2.23 (1.56)	1.9	0- 6.58
Meat & alternatives (1-2)	1.74 (1.39)	1.5	0-6.1
Other	2.99 (2.46)	2.5	0-11
Energy (kcal)	1734 kcal (650)	1666 kcal	389 – 4148 kcal

^1^Guidelines are based on the 2007 Eating Well with Canada’s Food Guide [32].

**Table 2:** **Socio-demographics of parents with children participating in the study**^**1**^

	**n**	**Percent of respondents %**
Were you born in Canada?	551	
1 = no	249	45.2
2 = yes	302	54.8
Education level	547	
1 = No schooling	25	4.6
2 = Elementary	103	18.8
3 = Secondary	208	38.0
4 = Community/Technical College	110	20.1
5 = University	101	18.5
Household income	375	
1 = Less than $25,000	37	9.9
2 = $25,000 - $50,000	88	23.5
3 = $50,001- $75,000	80	21.3
4 = $75,001 - $100,000	58	15.5
5 = More than $100,000	112	29.9

^1^Only valid percent displayed. Non-response rate for ‘Born in Canada’ is .2%, ‘Education’ is 8.2%, and ‘Household Income’ is 37.1%.

**Table 3:** **Descriptive statistics for responses from parents of children with baseline fruit and vegetable intake data**^**2**^

**Variable**	**Number**	**Percentage**
*I have good access to stores to purchase fresh fruits and vegetables*		
Strongly disagree	7	1.3
Disagree	13	2.4
Agree	201	36.2
Strongly agree	333	60.2
*How would you describe your child’s eating habits?*		
Unhealthy	10	1.8
Somewhat healthy	182	33.1
Healthy	302	54.9
Very healthy	56	10.2
*How many times each week does your family eat supper at the table together?*		
Never or < 1 time per week	27	4.9
1-2 times per week	56	10.2
3-4 times per week	123	22.4
5 or more times per week	344	62.5
*How many times each week does your family eat supper in front of the TV?*		
Never or < 1 time per week	329	60.4
1-2 times per week	121	22.2
3-4 times per week	47	8.6
5 or more times per week	48	8.8
*How many times each week does your family eat at a fast food restaurant, or eat food taken out from a fast food restaurant?*		
Never or < 1 time per week	350	63.5
1-2 times per week	191	34.7
3-4 times per week	10	1.8
5 or more times per week	none	none
*How much do you personally care about staying fit or exercising?*		
Not at all to a little bit	119	21.8
Quite a lot	253	46.3
Very much	175	32.0
*How much do you personally care about eating healthy foods?*		
Not at all to a little bit	43	7.9
Quite a lot	286	52.5
Very much	216	39.6

^2^Only valid percent displayed. Non response rate for; ‘Good access to FV’ is 7.2%, ‘How would you describe your child’s eating habits’ is 7.7%, ‘How many times does your child eat supper at the table together’ is 7.7%, ‘How many times each week does your family eat supper in front of the TV’ is 8.6%, ‘How many times does your family eat at a fast food restaurant’ is 7.6%, ‘How much do you personally care about staying fit’ is 8.2%, and ‘How much do you personally care about eating healthy foods’ is 8.6%.

### Correlates of SES and Social factors with fruit and vegetable intake

Relationships among all of the variables are shown in Table 4. Correlations between SES and the other social factors and fruit and vegetable intake are highlighted briefly below.

**Table 4:** Correlation matrix for SES and social factors with fruit and vegetable intake

	**FV intake**	**Born in Canada**	**Income**	**Education**	**FV access**	**Affluence**	**Neigh-bourhood composite score**	**Parent’s perception of child’s eating habits**	**Supper in front of TV (x/wk)**	**Supper at the table together (x/wk)**	**Eat out (x/wk)**	**Parent cares about fitness**	**Parent cares about eating healthy foods**
Fruit and vegetable intake	--	-.016	.038	.060	-.021	.089*	.089*	.115**	-.101*	.059	-.006	.055	.156**
Born in Canada	-.016	--	.261**	-.209**	-.040	.229**	.060	-.024	-.019	-.045	.019	.067	.007
Income	.038	.261**	--	.154**	.044	.384**	.268**	-.026	-.103*	-.020	-.016	.105*	.012
Education	.060	-.209**	.154**	--	.021	.140**	.052	.071	-.136**	.110*	-.012	.061	.041
Fruit and vegetable access	-.021	-.040	.044	.021	--	.087*	.241**	.089*	.024	-.047	.008	.069	.063
Affluence	.089*	.229**	.384**	.140**	.087*	--	.154**	-.003	-.043--	.050	-.015	.017	-.012
Neighbourhood composite score	.089*	.060	.268**	.052	.241**	.154**	--	.088	-100*	.057	.030	.180**	.147**
Parent’s perception of child’s eating habits	.115**	-.024	-.026	.071	.089*	-.003	.088	--	-.162**	.227**	-.157**	.116**	.225**
Supper in front of TV (times/wk)	-.101*.	-.019	-.103	-.136**	.024	-.143*	-.100	.162**	--	-.370**	.173**	-.033	-.048
Supper at the table together (times/wk)	.059	-.045	-.020	.110*	-.047	.050	.057	.227**	.370**	--	-.111**	.090*	.110*
Eat out (times/wk)	-.006	.019	-.016	-.012	.008	-.015	.030	.157**	.173**	-.111**	--	-.012	-.163
Parent cares about fitness	.055	.067	.105*	.061	.069	.017	.180**	.116**	-.033	.090*	-.012	--	.520**
Parent cares about eating healthy foods	.156**	.007	.012	.041	.063	-.012	.147**	.225**	.048	.110*	-.163**	.520**	--

*correlation is significant at the 0.05 level.

**correlation is significant at the 0.01 level.

All correlations are Spearman’s correlations except for Born in Canada, calculated using point-biserial correlation.

### Socio-economic status

Parent reported income and education level were not significantly related to children’s fruit and vegetable consumption (*r*_*s*_ = 0.04 p > 0.05 and *r*_*s*_ = 0.06, p > 0.05 respectively). Further, having a parent born in Canada was not significantly related to child’s consumption of FV (*r*_*pb*_ = -0.02, p > 0.05). Child reported Family Affluence was significantly related to FV consumption (*r*_*s*_ = 0.09, p < 0.05) but was below the lower limits for meaningfulness suggested by Cohen [42].

Although parent income and education were not significantly associated with child FV consumption they were associated with each other, child-reported family affluence, neighbourhood environment, access to FV, and eating at the table or in front of the television.

### Social factors

There was a weak but significant positive relationship between the neighbourhood composite score (M = 25.05, SD = 3.2) and children’s FV intake (*r*_*s*_ = 0.09 p < 0.05). The frequency of eating dinner in front of the television during the week was negatively related to FV intake (*r*_*s*_ = -0.10, p < 0.05) while parent’s perception of the healthiness of their child’s diet was positively correlated (*r*_*s*_ = 0.12, p < 0.01). How much a parent reported personally caring about eating healthy foods was also significantly related to their child’s FV consumption (*r*_*s*_ = 0.16, p < 0.01). How much they cared about staying fit and exercising, perceptions of access to healthy FV, and eating supper at the table or at a fast food restaurant were not significantly correlated to children’s FV consumption. A number of social factors and SES were correlated with each other (see Table 4).

### Multiple regression analysis

A multiple regression model (Table 5) was conducted with all variables that were significantly correlated with FV consumption (parent’s perceptions of their child’s eating habits, parent personally caring about eating healthy foods, the neighbourhood environment score, eating supper while watching television, FAS). Of the variables entered into the model, only FAS was significantly associated with FV intake although once again this relationship was very weak, almost trivial. A one standard deviation increase in FAS predicted increased intake of FV (std-β = 0.096, 95% CI = 0.01 to 0.27). While holding all other variables constant in the model, every 1.74 unit (1 standard deviation) increase in reported affluence was associated with an extra 0.17 servings of FV.

**Table 5:** Multiple regression results examining predictors of children’s fruit and vegetable intake

	**std-β**	**95% confidence interval**	**p-value**
*Family affluence score*	0.096	0.01 to 0.27	0.04
*Neighbourhood environment*	0.062	-0.02 to 0.12	0.17
*Perceptions of children’s eating habits*			
Unhealthy	Reference group	--	--
Somewhat healthy	0.105	-1.21 to 2.34	0.53
Healthy	0.264	-0.43 to 3.10	0.14
Very healthy	0.171	-0.49 to 3.24	0.15
*Supper in front of the television*			
Never or <1 time per week	Reference group	--	--
1-2 times per week	-0.056	-0.90 to 0.22	0.24
3-4 times per week	-0.014	-0.94 to 0.70	0.77
5 or more times per week	-0.078	-1.51 to 0.12	0.09

## Discussion

We set out to explore the associations between socio-economic and other social factors and fruit and vegetable consumption in pre-adolescent children in BC. First and foremost however, our findings echo previous research [23] that showed that irrespective of SES the majority of the children were failing to meet the recommended daily guidelines for fruit and vegetable consumption. The proportion below the recommendations in our study (85%) was even higher than the 62% of boys and 68% of girls who were below recommendations in the Canadian Community Health Survey, Cycle 2.2 (CCHS 2.2) [21]. This may be because the recommended minimum number of servings at the time the CCHS 2.2 was conducted was 5 servings per day, which was subsequently increased to 6 servings per day in the 2007 food guide [32].

Second our results provided little evidence to suggest that socio-economic status and other social determinants were associated with children’s fruit and vegetable consumption. Parent income is one of the primary indicators of socio-economic status, and it has been linked to higher intake of fruit and vegetables among children in some studies [12,15,23,26]. Counter to these findings parent income was not significantly correlated with fruit and vegetable intake among children in our study. Our result is supported by studies among Australian adolescents [14], 9 and 10-year-old American girls [20] and 4-18 year old Canadian children and youth [21]. Interestingly, child-reported affluence, which is a surrogate measure for income, was associated, albeit very weakly. This may reflect subtle differences in the measures. The affluence measure is a relative measure, most closely associated with the concept of disposable income, while parent-reported income is an absolute measure, not adjusted for items like family size, housing costs, debt, etc. Nevertheless, the finding was in the trivial range in terms of effect size so the deviations between income and affluence may be negligible in terms of predictive utility.

Based on our correlation results however, it is possible that there is a developmental aspect to the relationships we explored. For instance parental income might be related to fruit and vegetable consumption through a set of behaviours that may aggregate and take time to fully manifest as measurable fruit and vegetable consumption effects. This could explain the significant relationship between SES and consumption found in adolescence and then more consistently in studies of adults. In our study for example, lower-income parents were more likely to report eating dinner in front of the television, which itself was significantly – although again, weakly -- correlated with decreased fruit and vegetable consumption in our study and others [28]. Similarly parents with a higher income were more likely to report good access to fruit and vegetables. This relationship between income and access to fresh produce has been highlighted previously [43]. However, reported access did not correlate with children’s consumption in our study, which may reflect the level of access measured. The lack of relationship may be because our measure was about neighbourhood access while the determinants literature identified access in the home [4,27,28]. We also used ‘perceived access’ rather than directly measured access and thus our measure might reflect parental awareness and attitudes rather than actual access.

Education is another commonly cited indicator of social economic status [4,20,23,25,30]. In fact, education was positively associated with parent income in this study. Evidence has indicated that lower parental education status is associated with poorer diet quality, including higher fat and lower micronutrient intake in children [4,20]. In their study of Canadian adolescents, Riediger and colleagues reported a small but significant positive association between parental education and fruit and vegetable intake [23]. Our results did not support this direct relationship in pre-adolescent children. Instead, as with parent income, it appeared that relationship between parent education and fruit and vegetable intake had the potential to develop over time influenced by a set of lifestyle behaviours established during childhood. Specifically, lower parental education was associated with eating dinner at the table less frequently, and eating in front of the television more frequently. The latter was correlated with decreased fruit and vegetable intake in our study and in others [28].

The fact that parental income and education weren’t directly associated with fruit and vegetable consumption could also be related to measurement issues or other factors that play an important role in determining what children eat. Numerous studies have discussed factors associated with the dietary intake of children that are not directly related to socio-economic status [4,28,44,45]. We found that parental perceptions about the ‘healthiness’ of their child’s diet related positively to fruit and vegetable consumption. The direction of influence can’t be elucidated in a cross-sectional survey. We also found that the perceptions of the neighbourhood environment score, which is primarily a measure of liking for the neighbourhood and access to safe physical activity opportunities, was positively associated with the perceived ‘healthiness’ of the child’s eating habits. Parents who rated their neighbourhood highly were more likely to indicate that they thought their child’s eating habits were good, encourage their children to eat healthy foods, and personally care about eating healthy foods. Notably, both income and education were positively associated with the neighbourhood composite score. However, these findings should be placed in context by noting that the variables explored failed to predict a meaningful amount of the variability in children’s fruit and vegetable intake (small to trivial effect sizes) and that intake was below recommended amounts for almost all children.

Our findings should be considered in light of a number of limitations. One such limitation, present in almost all studies of nutrient consumption in children, is that in order to collect data efficiently from a large number of subjects (both financially and practically speaking), it is often necessary to rely on children’s self-report of their dietary intake; dietary self-reports rely on memory, which is subject to error. The 24-hour recall method is a well-studied method in dietary recall testing, particularly when assessing mean intakes of population groups [30], however, the ability of children to accurately recall and report their intake has been called into question in a number of studies [46-48]. We attempted to mitigate this limitation by measuring the oldest children in the schools.

Evidence indicates that while there are inherent difficulties in collecting nutritional data from large groups of children through the use of self-report surveys, if properly administered (i.e., the length of time between intake and report is minimized), such tests can give moderately accurate results, and are generally the most efficient and cost-effective method of collecting such data. However, this is likely true only if the children are ten years of age or older [49]. As the children in this study were between the ages of ten and twelve, with an average age of 11.3 years, it is probable that most of the participating children were of an age where they could accurately complete the surveys used in this study.

A second limitation, highlighted by the response rates and descriptive statistics, was a potential sampling bias. As with all studies we depended on volunteers and although it appears that the SES of the adult participants was close to the reported population norms in 2011 [50,51] our data showed that most reported caring about eating healthy foods. Accordingly, they may have been more likely to engage in health promoting behaviours than a randomly selected population would be. Finally, there are limitations inherent to cross-sectional designs and the fact that intake was assessed on only a single day and might not reflect a pattern of nutrient consumption established over a period of time.

## Conclusion

Canadian children in BC were not meeting Canada’s food guidelines for fruit and vegetable consumption irrespective of SES. Low fruit and vegetable intake appears to be a population wide concern. It is imperative that public health policy-makers and practitioners act to promote healthy eating, particularly of foods that are under consumed by large proportions of individuals, such as fruit and vegetables. This promotion, however, must be underpinned by a thorough understanding of the determinants of dietary behaviour. While socio-economic status is consistently associated with the nutritional status of adults, in BC we found only weak to trivial evidence that socio-economic factors relate to pre-adolescent children’s fruit and vegetable consumption. The home environment, parental perceptions and habits, were weakly associated with fruit and vegetable consumption and also with parental income and education and the influence of these and other factors deserves further exploration using longitudinal designs to explore the development of fruit and vegetable consumption behaviours over time.

## Abbreviations

FV: Fruit and vegetable; BC: British Columbia; FAS: Family Affluence Scale; SES: Socio-Economic Status; SFVNP: School Fruit and Vegetable Nutritional Program.

## Competing interests

The authors declare that they have no competing interests.

## Authors’ contributions

AA developed the research question, conducted the preliminary analysis and drafted the manuscript. PJN, wrote the funding proposal, managed the implementation of the overall study, supervised the analysis of the baseline data and edited the manuscript. RR and SB were Co-Investigators/co-authors on the funded grant proposal, contributed to research design, instrument selection and analysis and edited the manuscript. JS, managed the implementation of the project, entered and helped with analysis of the data and co-authored the manuscript. AY developed and implemented the data analysis using multiple linear regression, wrote the results section, and edited the manuscript. All authors read and approved the final manuscript.

## Pre-publication history

The pre-publication history for this paper can be accessed here:

http://www.biomedcentral.com/1471-2458/14/150/prepub
